# Extrapelvic endometriosis: a rare entity or an under diagnosed condition?

**DOI:** 10.1186/1746-1596-8-194

**Published:** 2013-12-02

**Authors:** Nikolaos Machairiotis, Aikaterini Stylianaki, Georgios Dryllis, Paul Zarogoulidis, Paraskevi Kouroutou, Nikolaos Tsiamis, Nikolaos Katsikogiannis, Eirini Sarika, Nikolaos Courcoutsakis, Theodora Tsiouda, Andreas Gschwendtner, Konstantinos Zarogoulidis, Leonidas Sakkas, Aggeliki Baliaka, Christodoulos Machairiotis

**Affiliations:** 1 Obstetric - Gynecology Department, ”Thriassio” General Hospital of Athens, George Genimata, 19600 Athens, Greece; 2 Surgery Department, “Thriassio” General Hospital of Athens, George Genimata, 19600 Athens, Greece; 3 Internal Medicine Department, General Hospital of Syros, Nikiforou Mandilara, 84100 Island of Syros, Greece; 4 Pulmonary Department, “G. Papanikolaou” General Hospital, Aristotle University of Thessaloniki, Exohi 1100, 57010 Thessaloniki, Greece; 5 1st Internal Medicine Department, “Thriassio” General Hospital of Athens, George Genimata, 19600 Athens, Greece; 6 Surgery Department (NHS), University General Hospital of Alexandroupolis, Nea Makri, 68100 Alexandroupolis, Greece; 7 Radiology Department, University General Hospital of Alexandroupolis, Nea Makri, 68100 Alexandroupolis, Greece; 8 Internal Medicine Department, “Theiageneio” Anticancer Hospital, Alexander Simeonidi 2, 54007 Thessaloniki, Greece; 9 Pathology Department, Hospital of Amberg, Mariahilfbergweg 5-7, 92224 Amberg, Germany; 10 Pathology Department, “G. Papanikolaou” General Hospital, Exohi 1100, 57010 Thessaloniki, Greece

**Keywords:** Endometriosis, Lung, Ovary, Molecular pathways, Treatment

## Abstract

Endometriosis is a clinical entity characterized by the presence of normal endometrial mucosa abnormally implanted in locations other than the uterine cavity. Endometriosis can be either endopelvic or extrapelvicdepending on the location of endometrial tissue implantation. Despite the rarity of extrapelvic endometriosis, several cases of endometriosis of the gastrointestinal tract, the urinarytract, the upper and lower respiratory system, the diaphragm, the pleura and the pericardium, as well as abdominal scars loci have been reported in the literature. There are several theories about the pathogenesis and the pathophysiology of endometriosis. Depending on the place of endometrial tissue implantation, endometriosis can be expressed with a wide variety of symptoms. The diagnosis of this entity is neither easy nor routine. Many diagnostic methods clinical and laboratory have been used, but none of them is the golden standard. The multipotent localization of endometriosis in combination with the wide range of its clinical expression should raise the clinical suspicion in every woman with periodic symptoms of extrapelvic organs. Finally, the therapeutic approach of this clinical entity is also correlated with the bulk of endometriosis and the locum that it is found. It varies from simple observation, to surgical treatment and treatment with medication as well as a combination of those.

**Virtual slides:**

The virtual slide(s) for this article can be found here: http://www.diagnosticpathology.diagnomx.eu/vs/1968087883113362.

## Introduction

Endometriosis is defined as the presence of normal endometrial mucosa abnormally implanted in locations other than the uterine cavity. Depending on the area identified, endometriosis is characterized as endopelvic or extrapelvic [1-8]. The endopelvic ectopic implants are located in the minor pelvis, the ovaries, the fallopian tubes and the uterosacral ligaments posterior of the uterus, whereas, the more unusual extrapelvic implantation sites are the abdominal wall, scars of the perineum, the urinary and gastrointestinal tract, the thorax and the nasal mucosa. Endometriosis can affect any woman from promenarche until postmenopause, regardless the race or ethnicity and her maternal status [9].

Endometriosis is the most common cause of chronic pelvic pain in females. Its prevalence has been estimated to 1-2% of reproductive age females and its more common (15-25%) among women with infertility problems [10]. The prevalence is 40-60% among women with dysmenorrhea and it is extremely rare after menopause, because of the estrogen dependence of the ectopic tissue [10]. The relapse of endometriosis during menopause has been correlated with hormonal replacement therapy [11].

Endometriosis is not related with endometrial cancer. Current research has demonstrated a relationship between endometriosis and certain types of neoplasms mainly ovarian cancer, non-Hodgkin lymphoma and brain cancer [12,13]. Endometriosis often coexists with fibroid or adenomyoma, and autoimmune disorders. A survey conducted in 1988 in the U.S. found significantly higher prevalence of cases of hypothyroidism, fibromyalgia, chronic fatigue syndrome, autoimmune diseases, allergy and asthma in women with endometriosis compared to the general population [14].

### Mechanism of the pathogenesis of endometriosis

Endometriosis is one of the most enigmatic and mysterious gynecological diseases [15]. The etiology and pathogenesis, knowledge of which is the basis of dealing with it, still remains very unclear. Since 1860, when Von Rokitanski [16] made the first detailed description of this condition, many theories have been developed for the etiology and pathogenesis of endometriosis, but none fully meets the pathological entity. The first thoughts about the etiology-pathogenesis of endometriosis were made in 1885 by Von Recklinghausen, who considered that embryonic mesonephric elements are responsible for the development of endometriosis [17]. The view of Von Recklinghausen was adapted in 1942 by Grunwald who argued that during the fetal cells from the growing resources Muller can acquire the ability to convert them into endometrial cells [18].

Currently three theories seem to represent the current scientific thinking. These are the theory of metaplasia (metaplasia theory), the theory of dispersion and ‘transplantation’ of endometrium and the theory of induction. It is of great importance to refer that these three theories are the “core” of the current scientific thinking and there are many elements added to this core creating a variety of theories. On the other hand, nowadays, as a result of extensive literature research there is scientific evidence questioning the existence of endometriosis as a single entity and the accuracy of the theories above.

The first theory is the theory of metaplasia of the peritoneal serosa cuff. This theory was proposed by Meyer in 1903 [17]. According to this theory, the development of the disease is a result of a continuous process of tissue differentiation of mesothelial cells of the peritoneum under the effect of inflammatory or hormonal factors, resulting in the formation of cell clusters which gradually sink to the underlying organs while they are reshaping in endometrial glands and stroma. This theory that found great popularity in the early decades of the twentieth century could explain the development of endometriosis in women with agenesis resources Muller. But there are several problems and questions, which cannot be answered by this theory. However, the theory of metaplasia, can explain the development of endometriosis in distant organs.

The second theory of the aetiology and pathogenesis of endometriosis is that of endometrium dispersion and ‘transplantation’. The dispersion of endometrial cells through the lymphatic vessels was proposed by Halban on 1924 in order to interpret the extrapelvic localizations of endometriosis [18]. The hematogenous spread was supported on the same basis by Sampson on 1925 [19]. Histologic confirmation of this theory was made by Javert on 1952, who proved the presence of endometrial tissue in pelvic veins [20]. The mostly understood and accepted theory is the theory of dispersion proposed in the 1920s by Sampson, who supported that during the menstruation, retrograde dispersion of living endometrial cells can occur in the peritoneal cavity and ovaries.

The third theory is that of induction, which can be considered as a combination of the two previous theories [21]. According to this theory, unknown substances, are released from the endometrium and cause transformation of undifferentiated mesenchymal cells to endometrial tissue. This theory is supported experimentally by Merril, which caused peritoneal endometriosis in rodents, implanting in the peritoneal cavity filters of live endometrial tissue [22].

Most of the modern theories of endometriosis pathogenesis consist of the following additional areas:

*Estrogens:* Endometriosis is a condition that is estrogen-dependent and therefore is considered to occur mainly during the reproductive age [10]. In experimental models, estrogens are necessary in order to induce or preserve endometriosis. The treatment often aims at the reduction of the estrogen levels in order to control the disease.

*Genetics:* Hereditary factors appear to play a role. It is known that the daughters or sisters of patients with endometriosis are at higher risk of developing endometriosis themselves. For example, the low levels of progesterone may have a genetic etiology, and can contribute to a hormonal imbalance. There is an approximately 10-fold increase in the disease incidence in women with affected first-degree relative [23]. A 2005 study published in the American Journal of Human Genetics has shown an association between endometriosis and chromosome 10q26 [24], while a 2010 study in Nature Genetics identified association with the region 7p15.2 [25]. One study found that women-siblings of patients with endometriosis, the relative risk for endometriosis is 5.7:1 versus the general population [26].

*Transplantation:* It is widely accepted that in specific patients endometriosis can spread directly. Thus, ectopic endometrial tissue is found in abdominal surgical scars after surgery for endometriosis [9].

*Immune System:* The research focuses on the possibility that the immune system may not be able to cope with retrograde menstruation. In this context, there is interest in studying the relationship between endometriosis and autoimmune disease, allergic reactions, and action of toxins [27,28]. It is still unclear what, if any, the causal relationship among toxins, autoimmune disease, and endometriosis is.

*Context:* There is a growing suspicion that environmental factors may cause endometriosis, specifically some plastics and cooking with certain types of plastic containers with microwaves [29] and the effect of dioxin [30,31]. Other sources indicate that pesticides and hormones in our food cause a hormonal imbalance.

*Congenital defect:* In rare cases where the atretic hymen is not terminated before the first menstruation and remains trapped inside the patient’s uterus until the problem is resolved with surgical resection. Many health care professionals do not address this flaw, and often overlooked until multiple menstrual periods to spend.

However, the most widely accepted theory for the pathogenesis of endometriosis is the theory of *ectopic endometrial implantation* via retrograde menses [32]. Similarly, endometriosis in distant parts of the body can be explained by the migration of cells of the endometrium through lymphatic and blood vessels [33]. Thus, endometriosis is a unique example of benign proliferation and metastasis [34].

At cellular level, endometriosis is characterized by development of monoclonal tissue and may have characteristics of malignant behavior, including local and metastatic filtration. As a result of this endometriosis can be used as a model to study the molecular and genetic conditions needed for dispersion or non-malignant cells.

Generally, the correlation between endometriosis and cancer is unclear. Although endometriosis is a non-neoplastic disease, and does not cause catabolic syndrome and cahexia, other procedures, which characterize the carcinogenesis and metastasis are also present in endometriosis. These include cell motility, cell adhesion and infiltration, immunological factors, maintaining the original structure and architecture of the tissues in ectopically points, angiogenesis and metaplasia [35].

Furthermore, there is evidence that endometriosis has genetic alterations similar to some cancer types [35,36], while a direct correlation between ovarian cancer and endometriosis has been previously described in some reports and clinical trials [13,37-39]. Like any other tissue, endometrial tissue can also undergo malignant transformation. On the contrary, large retrospective epidemiological studies suggest that women with endometriosis have an increased risk of ovarian and some other cancers compared to the general population [40-42].

#### The role of the extracellular matrix enzymes in endometriosis

A series of studies has shown that several enzymes of the extracellular matrix act to endometriosis or in normal endometrium of women with endometriosis, leading to self-destruction of the extracellular matrix. Thereby, facilitating the penetration of epithelial cells in deeper layers of the endometrium, which are required either for local development of endometriosis foci or disease spread. Of the various enzymes of the extracellular matrix which act in this manner have been studied mainly the system of metalloproteinases and the plasminogen system.

The system of metalloproteases or matrix metalloproteinase (MMP-matrix metoloproteases or metalloproteinases) comprises an enzyme component, the MMPs, and by an inhibitory element of enzymes, the tissue inhibitor of metalloproteases the TIMPs (tissue inhibitor of metaloproteases) [43]. It is well documented that the system of MMPs plays a key role in the normal development and growth of the endometrium and many other physiological processes in other tissues. Because of the necessity for balance between MMPs and TIMPs, it is not surprising that a differential expression of MMPs and TIMPs is associated with the pathophysiology of endomiolysis and the imbalance between the secretion of MMP-9 and its natural inhibitor TIMP-1 in the culture milleu of endometriosis tissue probably reflects the increased ability of this tissue in vivo to cleave the extracellular matrix, thereby facilitating the ectopic implantation of endometrial growth.

The potential role of MMP-9 and TIMP-1 in the pathogenesis of endometriosis has also been studied in the peritoneal fluid of women with endometriosis by Stamatowicz et al. [44]. The findings of this study also agree with the hypothesis that the imbalance between MMP-9 and TIMP-1 may play an important role in the pathogenesis of the disease.

The Gaetje et al. [45] studied the expression of MMP-5-type membranes with microarrays and real time PCR. This study showed that the endometrium, and this part of the system of matrix metalloproteases contribute in an increased tissue remodeling (tissue remodeling) and allow cell migration in patients with endometriosis.

The Gillabert-Estelles et al. [46] studied the expression of angiogenic factors in endometriosis and their relationship with the system of matrix metalloproteinases. In this study, there was a significant increase in the peritoneal fluid of women with endometriosis, the levels of vascular endothelial growth factor (Vascular Endothelial Growth Factor-VEGF), the urokinase-type plasminogen activator (urokinase-type Plasminogen Activator-uPA) and the levels of MMP-3 in comparison with women-witnesses without endometriosis.

The MMP-3 and uPA were also studied by Ramon et al. [47] using quantitative realtime RT-PCR. In this study, there was an increase in the levels of uPA and MMP-3 in the endometrium of women with endometriosis, which can facilitate the adhesion of endometriosis tissue in the peritoneum and the surface of the ovary, and the penetration of the extracellular matrix, with the last one resulting in the formation of early endometriosis foci.

Another study, by Gillabert-Estelles et al. [48], reached similar conclusions. In this work, the scientists analyzed the expression of different members of the plasminogen activator and MMPs systems of endometriosis and found that the ovarian endometriosis tissues had higher levels of inhibitor of plasminogen activator 1 (Plasminogen Activator Inhibitor-PAI-1) and TIMP-1, compared to normal endometrium.

The overall conclusion is that the increase of uPA and MMP-3 in the endometrium of women with endometriosis leads in an increase in the invasive capacity of endometriosis cells. Since endometriosis creates a corner an increase in PAI-1 and TIMP-1 is detected, resulting in the cessation of further proteolytic activity.

The growth stop and the decrease in the proteolytic activity could also explain the frequent clinical finding of isolated endometriosis cysts without infiltration of the surrounding ovarian tissue.

In another study, Chung et al. [49] analyzed the expression of MMP-9 and TIMP-3 in normal and ectopic endometrium of women with endometriosis. Their findings show that both ectopic and normal endometrium of patients with endometriosis, may be more invasive and more capable of being implanted in the peritoneum because of the increased expression of MMP and decreased expression TIMP-3 in comparison with women without endometriosis.

The expression of another member of the MMPs family, the MMP-2, analyzed by Kim et al. [50] together with the expression of CD44s, of VEGF and Ki-67 in peritoneal, and ovarian endometriosis. In red and white endometriosis, there was a higher expression of MMP-2 in layer than in black homes. Also, the expression of MMP-2 was significantly elevated in advanced endometriosis (stages III and IV according to the revised classification of the American Fertility Society), indicating that MMP-2 may be responsible for the development of endometriosis. Similar findings in terms of the MMP-2 and VEGF were also present in an experimental model in mice [51].

Like the other members of the family of MMPs, so the MMP-1 appears to play a role in the mechanisms of local perfusion. The Hudelist et al. [52] noticed that interleukin-1 and MMP-1 are increased in ectopic endometrium of patients with endometriosis, suggesting their involvement in the pathogenetic mechanisms that lead to local invasion and tissue destruction.

**Figure 1:**
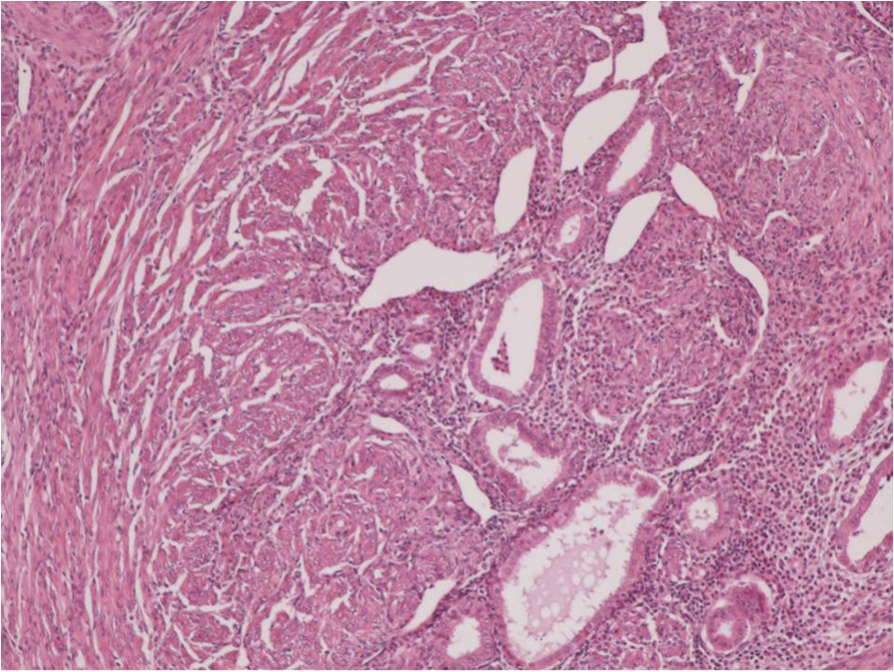
Clusters of endometrial glands and stroma in fallopian tube wall with inflammation (H&E X100).

**Figure 2:**
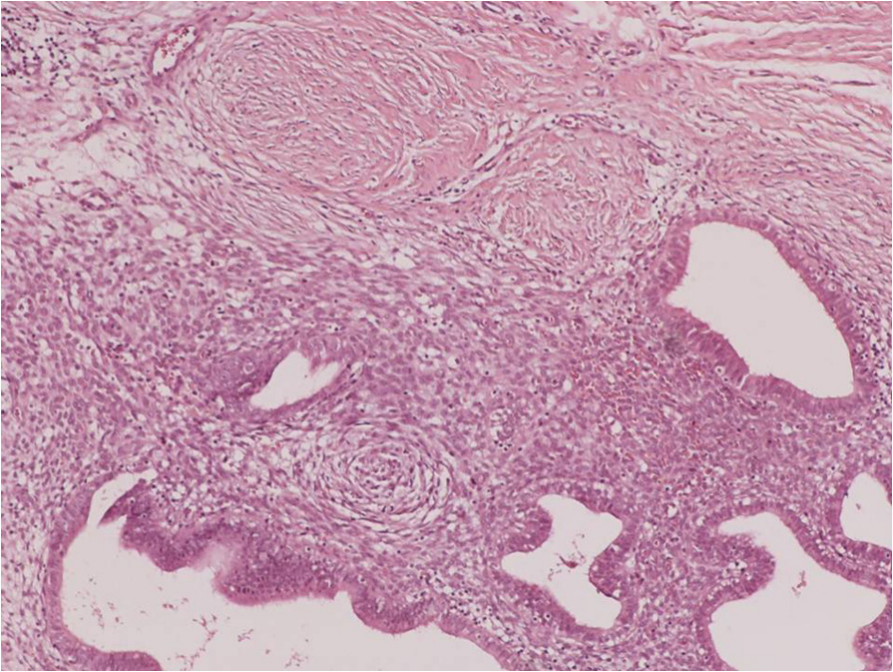
Nests of endometriosis into the rectus abdominis muscle (H&E X100).

**Figure 3:**
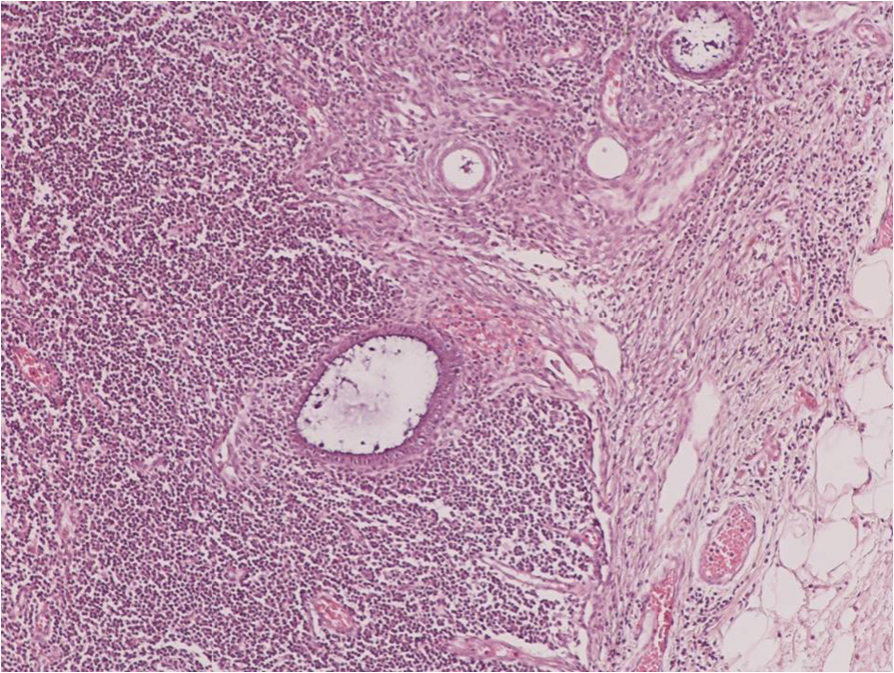
Focal endometriosis in a lymph node (H&E X100).

**Figure 4:**
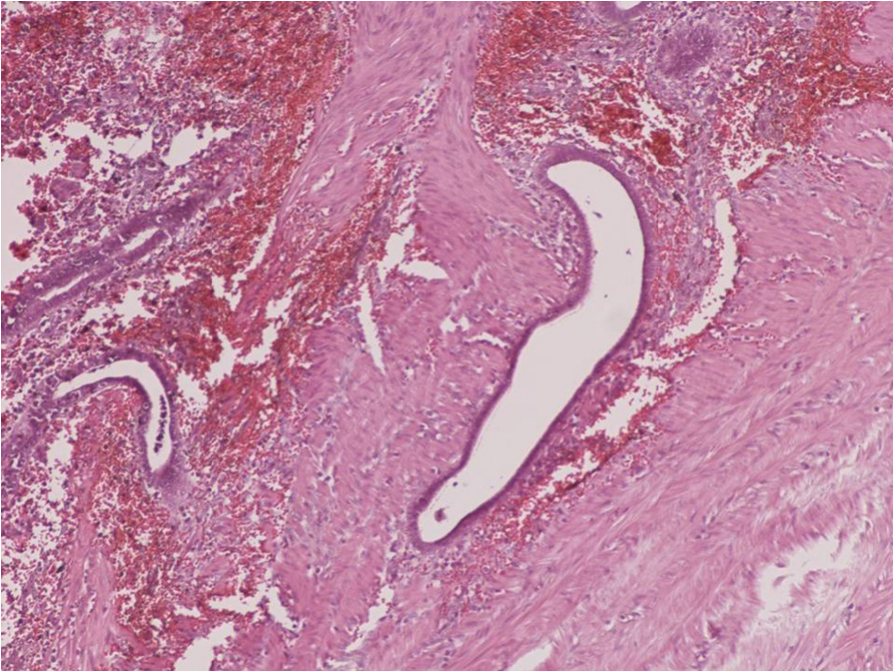
Endometrial glands and stroma with hemorrhange and hemosiderin-laden macrophages into the muscularis propria of large bowel (H&E X100).

**Figure 5:**
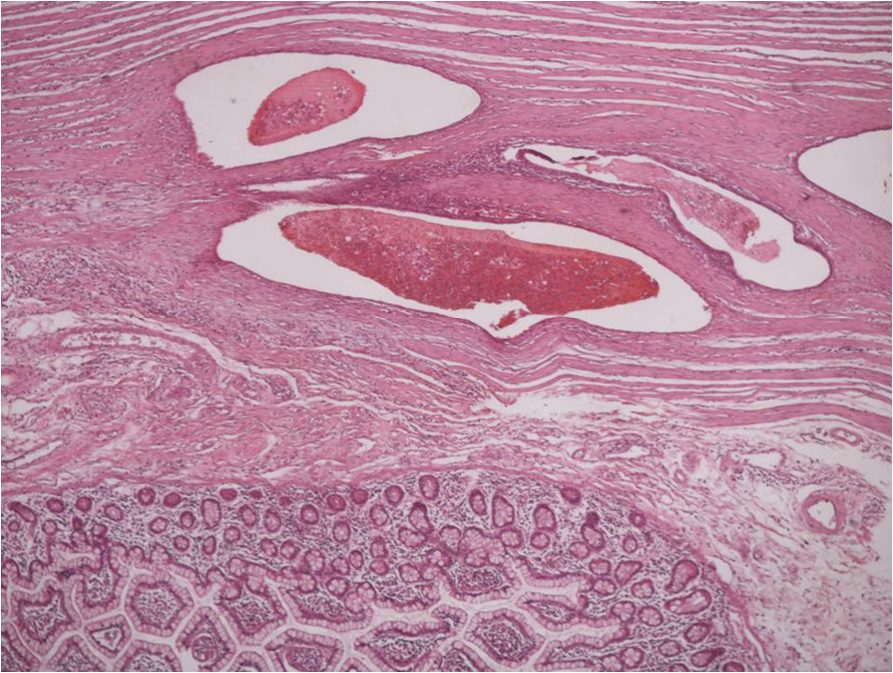
Nests of endometriosis into the muscularis propria of small bowel (H&E X40).

**Figure 6:**
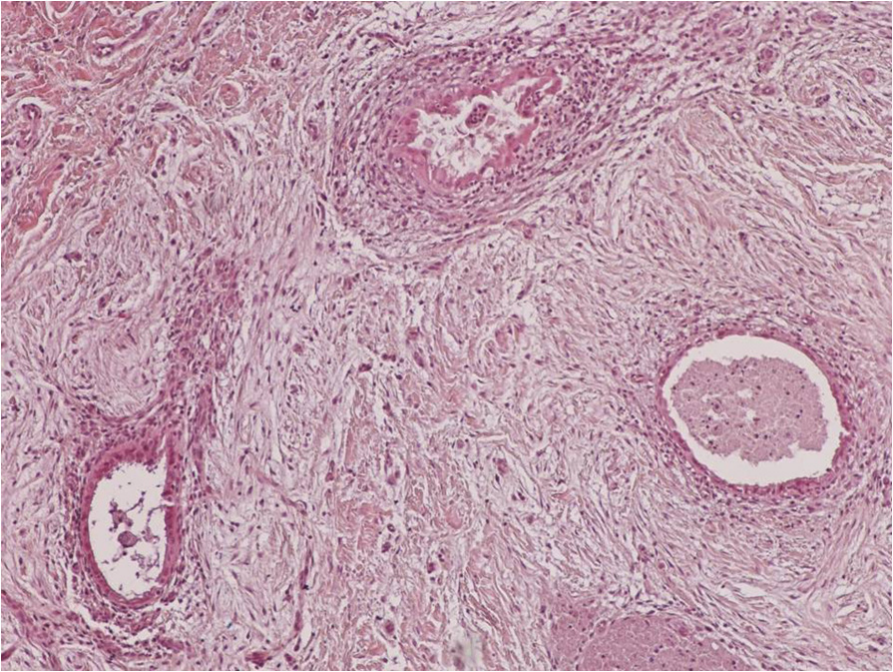
Endometrial glands and stroma into the dermis, close to cesarean section scar (H&E X100).

**Figure 7:**
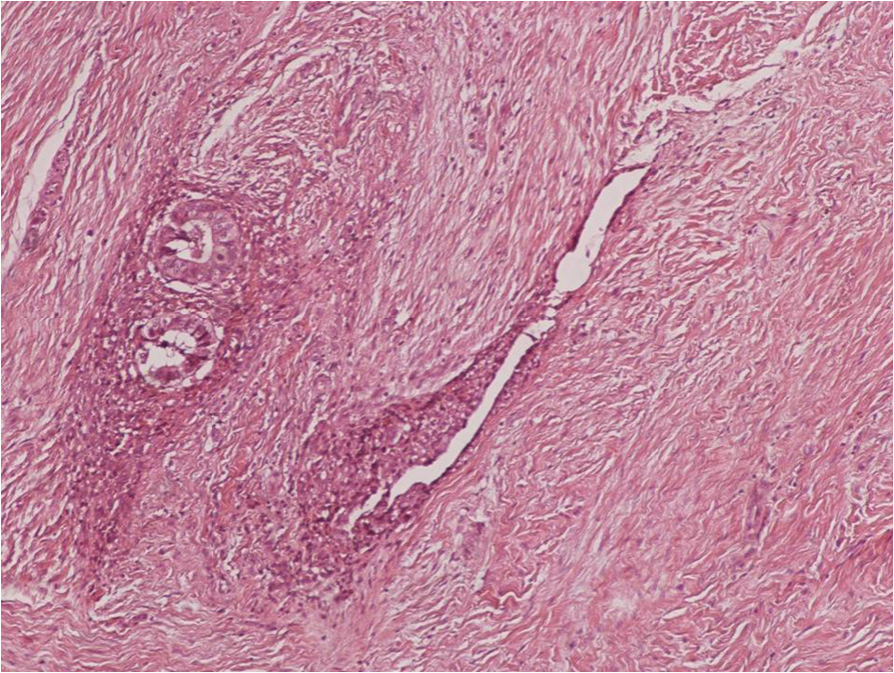
Nests of endometriosis in a fibrous backround, close to cesarean section scar (H&E X100).

Summarizing the findings of the literature, it is clear that the family of matrix metalloproteinases (MMPs) and their natural antagonists, inhibitors of metalloproteases (TIMPs), and the plasminogen system play an important role in the pathogenesis of endometriosis. However, it is still necessary, to conduct a substantial research effort to identify molecules which are more important in the mechanism of creation and development of endometriosis, and discovery of molecules which would be more suitable as markers of disease and as targets in which therapeutic models (Table 1) (Figures 1, 2, 3, 4, 5, 6, 7).

**Table 1:** Molecular pathways of endometriosis

**Past theories**	
Von Recklinghausen (1885)	Embryonic mesonephric elements
Grunwald (1942)	Fetal cells of Muller resources can be converted into endometrial cells
Metaplasia theory (Meyers 1903)	Metaplasia of the peritoneal serosa cuff differentiation of mesothelial cells of the peritoneum
Theory of dispersion and transplantation (Halban, Sampson)	Dispersion of endometrial cells through lymphatic vessels and hematogenous spread
Theory of induction (Merril)	Combination of the previous theories
**Modern theories combine**	
Estrogens	Estrogen dependent
Genetics	Hereditary (10q26, 7p15.2)
Transplantation	Direct spread
Immune system	Incapability of the immune system
Context	Environmental factors
Congenital defect	Atretic hymen
Ectopic endometrial implantation via retrograde menses	
**Cellular level**	Development of monoclonal tissue with characteristics of malignant behavior
**Molecular level**	Enzymes of the extracellular matrix act to endometriosis or in normal endometrium of women with endometriosis
MMP-9/TIMP-1	Stamatowicz et al.
MMP-9/TIMP-3	Chung et al.
MMP-5 type membranes increase	Gaetje et al.
MMP-3/uPA	Ramon et al.
VEGF/MMP-3/uPA	Gillabert –Estelles et al.
VEGF/MMP-2/CD44/Ki67	Kim et al.
PAI/TIMP-1	Gillabert –Estelles et al.
IL-1/MMP-1	Hudelist et al.

### Diagnostics of endometriosis

Some laboratory and imaging methods may help to diagnose the disease, but none of them alone is enough to diagnose endometriosis. Most often the diagnosis is made based on a combination of typical symptoms and clinical findings. Studies have shown that the diagnosis of endometriosis is earlier in women who are tested for sterility than in those with pelvic pain, in which the disease is diagnosed in more advanced stages, mainly due to the late visit to a gynecologist [9,53].

The main diagnostic methods for diagnosis of endometriosis are:

*Ultrasonography:* From the available imaging techniques, ultrasound has been proven useful in the diagnosis of endometriosis.

*Magnetic Resonance Tomography (MRI):* In controversial cases can confirm the diagnosis of endometriosis and to rule out other diseases.

*Diagnostic laparoscopy* with biopsy tissue remains the most reliable diagnostic method.

The only way to diagnose endometriosis is by laparoscopy or other types of surgery including biopsy of the lesion. The diagnosis is based on the characteristic appearance of the disease, and should be confirmed by biopsy. The surgery also allows for diagnosis of the surgical treatment of endometriosis at the same time [53,54].

### Biochemical markers

One area of diagnostic research is the search for markers of endometriosis. These markers are substances produced by or for the treatment of endometriosis and doctors can measure in biopsies, blood or urine. The detection of such an index can lead to early diagnosis of endometriosis that can be achieved by the rather non-specific symptoms, and can replace the invasive surgical procedures in the diagnosis of disease [55]. A biomarker could also be used to identify the first signs of therapeutic efficacy or recurrence of disease, because the symptomatic relief or deterioration is usually difficult to quantify [55]. However, since the benefits of the treatment of women with asymptomatic endometriosis are unclear, it is likely that any biomarker could be used only for the screening of women with symptoms suggestive of endometriosis [55]. Therefore, a predictive biomarker should distinguish between women with endometriosis than women with similar symptoms (for example, dysmenorrhea, pelvic pain or infertility [55].

A systematic study in 2010 for all proposed biomarkers of endometriosis in serum, plasma and urine concluded that none of them have been clearly shown to be of clinical use, although some seem to be promising [55]. Another study in 2011 identified several biomarkers supposedly after biopsy, including small sensory nerve fibers or reduced expression of b3 integrin subunit [56].

A biomarker has been used in clinical practice in the last 20 years is the CA-125 [13]. However, the performance in the diagnosis of endometriosis is low, but it seems to have an effect on the detection of more severe disease [55]. The CA-125 levels seem to fall during the treatment of endometriosis, but do not show any correlation with the therapeutic response [55].

Further research is also conducted for other potential genetic markers related to endometriosis that can replace surgical procedures for basic diagnosis [56].

Finally, a group of several biomarkers has been suggested as a future diagnostic tool for endometriosis, including both the concentrations of substances and genetic predisposition [55].

### Abdominal wall endometriosis

Abdominal wall is the most frequent location of extrapelvic endometriosis [57]. Endometriosis of the abdominal wall is usually associated with a surgical procedure in the uterus especially in women who have been delivered a cesarean section scar [58-63]. The most common clinical symptom in women with abdominal wall endometriosis is a constant focal abdominal pain which is mostly not associated with the menstrual cycle [59-62]. This pain is frequently atypical so it can be misdiagnosed. Patients can also feel a papable mass in the area of the surgical section.

Diagnostic methods for showing an abdominal endometrioma are: ultrasound sonography, computer tomography and magnetic resonance tomography (MRI).

Ultrasound sonography is not specific diagnostic method for an abdominal endometrioma, thus it shows a mass in the abdominal wall. This mass is imaged as a solid, hypoechoic lesion containing internal vascularity and it may also contain cystic areas. This abdominal mass must be submitted to differential diagnosis. This differential diagnosis includes neoplasms (like sarcoma or lymphoma), suture granuloma, ventral hernia, abscess or haematoma [64,65]. The latter three can be excluded from the final diagnosis of endometrioma by ultrasound sonography.

**Figure 8:**
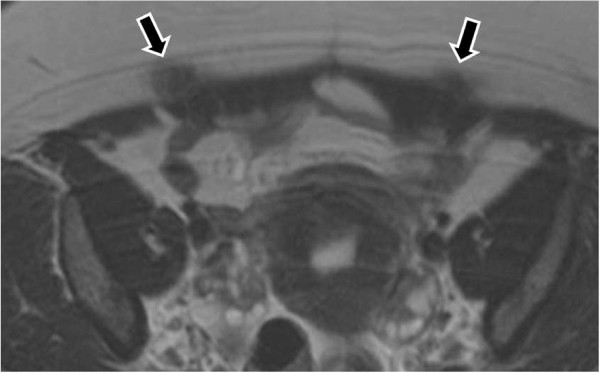
MR images demonstrate foci of endometriomas at the sites of the section [T-2 w.i].

**Figure 9:**
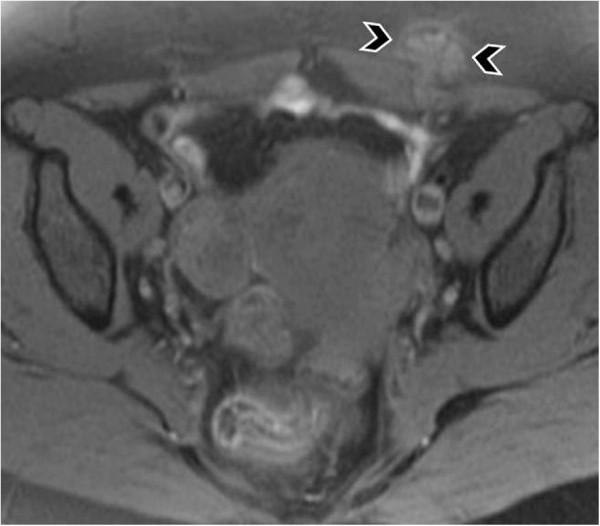
Endometrioma in the abdominal wall at point of the cesarian section [T-1w.i. and fat saturation technique].

**Figure 10:**
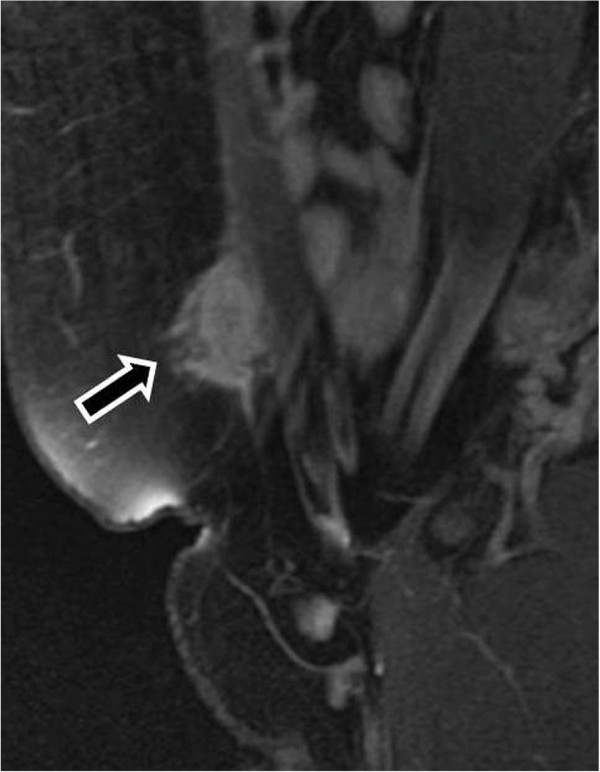
Endometrioma after enhanced T-1 w.i. and fat saturation technique.

CT and MRI also show a solid mass in the abdominal wall, so they cannot be specific diagnostic methods of an endometrioma, but they can depict the extent of the disease preoperatively [66,67] (Figures 8, 9, 10).

Though, sonographicaly guided FNA seems to be the most accurate diagnostic method for women with abdominal wall masses. Nevertheless, additional histological biopsy may be performed.

The best treatment of abdominal wall endometriosis is the wide surgical excision [9,58].

### Endometriosis of the thorax

Endometriosis of the thorax is a clinical entity that includes the presence of ectopic endometrial tissue in the pleura, the pericardium and rarely the diaphragm. This is often expressed as catamenial pneumothorax. Catamenial pneumothorax is the most common clinical expression of thoracic endometriosis syndrome, which includes four other entities. These are in brief, catamenial hemothorax, catamenial hemoptysis, endometriotic lung nodules, and catamenial chest pain. The catamenial character of all these symptoms mentioned above is a result of the menstrual cycle [68].

More specifically, for the development of catamenial pneumothorax the presence of endometrial tissue in the thoracic cavity is necessary. There are many theories trying to explain this phenomenon. The theory of Suginamy et al. suggests that endometrial tissue may circulate along with the peritoneal fluid in the abdominal cavity following a circle “route” down the left peritoneal gutter over the pelvic floor and up the right gutter to the peritoneal surface of the diaphragm. This “route” explains the increased frequency of catamenial pneumothorax of the right side [69].

The next question that rises after the description of this scenario is how these endometrial particles reach the pleura and the lung. Despite the fact that there are small peritoneal stomata, which allow the trespassing of particles below 30 Îm, to enter the diaphragmatic lacunae, a defect of the anatomical continuity of the diaphragm must also exist in order to allow air to pass and cause pneumothorax [70].

Another theory of the pathogenesis of catamenial pneumothorax was announced by Kirschner et al., who named it “porous diaphragm syndrome”. According to this theory preexisting diaphragmatic lesions allow fluid and gas to traverse the diaphragm and that the common development of pneumothorax in the right side is a result of the presence of the liver and the valve effect that it can cause to the intraperitoneal pressure [71].

**Figure 11:**
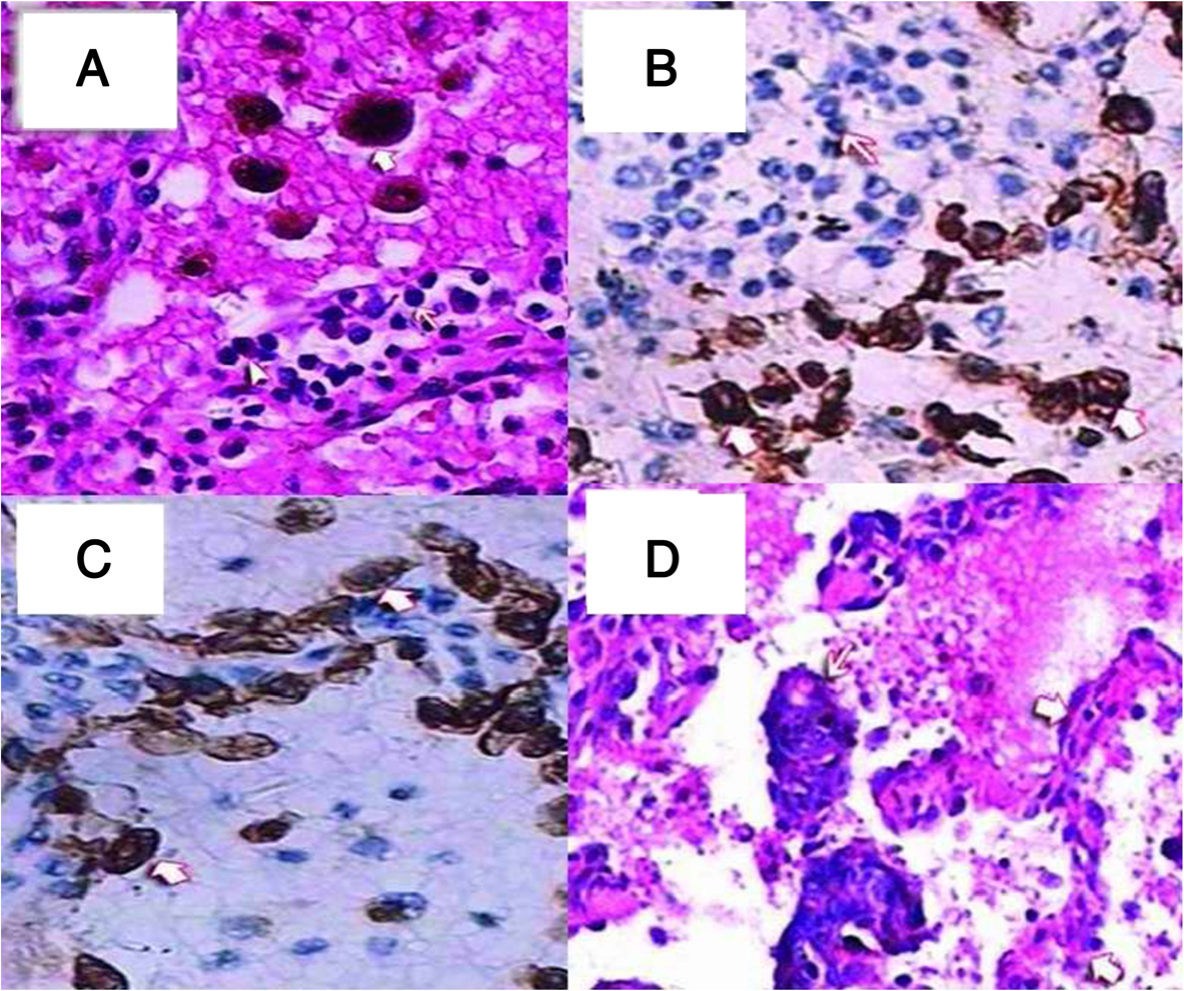
**Hematoxylin and eosin (H&E). A**: The alveolar spaces were filled with many red blood cells and phagocytic cells with hemosiderin (heavy arrowhead). The alveolar walls were infiltrated by plasma cells (triangulate arrowhead) and lymphocytes (light arrowhead); 200×. Immunohistochemical staining, **B**: Gland epithelium in an alveolus, CK7+ (heavy arrowhead). Infiltrating plasma cells and lymphocytes in the alveolar wall, CK7- (light arrowhead), H&E 200×, **C**: Phagocytic cells in the alveolar space, CD68+ (heavy arrowhead). Plasma cells and lymphocytes in the alveolar walls, CD68- (light arrowhead), H&E 200×. **D**: Atypical tubular-gland structures of decidual lesions were detected in the alveolar space (light arrowhead). Structure of alveolar wall (heavy arrowhead); 100×.

The other four clinical entities that form the thoracic endometriosis syndrome such as catamenial hemoptysis, catamenial hemothorax, lung nodules and catamenial chest pain can be a result of the lung lesions caused by the metastatic spread of endometrial tissue. In fact endometrial cells have been shown to embolize peripheral blood vessels of the lung as well as to invade the respiratory epithelium [70]. Another cause of catamenial hemoptysis can be the mensessynchronous increase of prostagalandine F2, which may cause rupture of bullae and blebs that may exist in normal lungs [70]. Additionally, to the cases of endometriosis of the lower respiratory system, there are two case reports in the literature tha refer to upper respiratory system endometriosis and more specifically to nasal endometriosis. Nasal endometriosis causes cyclic epistaxis and nasal pain, which is synchronous to the menstrual cycle [72] (Figure 11).

### Endometriosis of the gastrointestinal tract

Extrapelvic endometriosis can also be located in the liver and the gallbladder, but these entities are extremely rare. There are approximately fourteen cases in the international literature about liver endometriosis and in most of them the patients were suffering from pain and a feeling of weight in the right upper quadrant of the abdomen. There are also cases of liver endometriosis that was present with the clinical expression of obstructive jaundice [73-77].

Endometriosis of the gallbladder is extremely rare. There are two case reports in the literature referring to the diagnosis of gallbladder endometriosis [78].

In some women endometriosis occurs in the gastrointestinal tract. This is called intestinal endometriosis. The common endometriosis bowel symptoms are the rectal bleeding and pain, the painful bowel movements, the loss of appetite, the cramping stomach pains, the nausea and vomiting, the constipation and/or diarrhea, the abdominal bloating and gas in the abdomen. All these symptoms are getting worse during menstruation [79-82].

The most common location of extrapelvic intestinal endometriosis is the last part of the ileum (the small intestine), the cecum (the first part of the large bowel), and the appendix [83].

### Urinary tract endometriosis

Endometriosis of kidney is a rare condition. The common symptoms of renal endometriosis are local pain and rarely cyclical hematuria. It usually comes suddenly as a clinical manifestation. Sometimes the lesion may be totally asymptomatic and may diagnosed after nephrectomy for presumed renal cell carcinoma [84,85].

In ureteral endometriosis, ureteral involvement is often limited to one ureter, most commonly the left. Two major pathological types exist: extrinsic and intrinsic ureteral endometriosis. In the most common extrinsic type endometrial glandular and stromal tissue and the adventitia of the ureter or surrounding connective tissues are involved. In the intrinsic type muscularis propria, lamina propria, or ureteral lumen are involved [86,87]. Ureteral endometriosis can lead to urinary tract obstruction with subsequent hydroureter and hydronephrosis and even loss of renal function which is rare [88].

### Rare locations of endometriosis

Among the other rare locations of endometriosis it is important to refer the endometriosis of large muscles such as the adductor compartment [89], the endometriosis of the rectus abdominis muscle [90], endometriosis of the gluteal muscle, which can be cause catamenial sciatica [91]. There is one case report in the literature about nerve endometriosis. More specifically, it was endometriosis of the L5 nerve that caused gluteal atrophy and sciatica [92].

### Treatment

The therapeutic options in the treatment of endometriosis depend on the extent of the disease, the patient’s needs and the desire to maintain the reproductive capacity. These options include:

1) simple observation,

2) surgical treatment,

3) medical treatment and

4) combined therapy.

#### Simple observation

In the past the simple observation without any intervention was considered appropriate for the initial stages of the disease when no symptoms or are simply limited. Today therapeutic intervention after diagnosis of endometriosis is necessary as the lesions of the disease increases with time [93].

#### Conservative surgical treatment

Apply to patients who wish to preserve their reproductive capacity and may include removal or destruction (evaporation laser, electrocautery, thermal coagulation) surface lesions or endometriomas (cysts), which combined with cleaning of adhesions and restoring normal anatomy. The surgical treatment of the disease is either laparoscopy or laparotomy (open surgery). Regardless of the surgical technique (laparoscopy or laparotomy), removal of all endometrial lesions with careful cleaning of adhesions is necessary to address the pelvic pain and infertility in women who wish to preserve their reproductive ability [10].

#### Drug treatment

Theoretically, drug treatment would be ideal in the treatment of endometriosis. In practice, however, drug therapy alone is accompanied with a temporary improvement of pain, and tampering of the symptoms but in a time period they usually return. Also, drug therapy can certainly reduce the size of endometriomas and facilitate their removal surgery. Contraceptive pills are one of the main drug treatments, causing reduction of the quantity of blood and in that way they result in reducing pain during menstrual period. Additionally, Gonadotropin-releasing hormone (GnRH) agonists block the production of ovarian-stimulating hormones, lowering estrogen levels and preventing menstruation. This causes endometrial tissue to shrink. GnRH agonists can force endometriosis into remission during the time of treatment and sometimes for months or years afterwards The effectiveness of drug therapy on the reproductive capacity is questionable. That is the reason why drug therapy is not recommended as the sole treatment of women with endometriosis, except for rare cases where surgery is not possible or presents a significant risk for the life of the patient [94-96].

#### Combination therapy

The most effective way of treating endometriosis is the combination of surgical removal of all visible endometrial lesions and medication [97,98].

## Conclusion

Endometrium is one of the most extraordinary tissues of the human body. The ability of endometrium to be implanted in different tissues and simultaneously to maintain its functionality is very impressive. It also explains the variety of symptoms that are components of endometriosis syndrome. Pain is the main but not the only and not the pathognomonic characteristic of endometriosis [53]. Only the catamenial character of the symptoms can be considered as more indicative of this entity. In conclusion, endometriosis is a common clinical entity even in its extrapelvic form. Every clinician should have a high suspicion if it in cases of women with periodical symptoms [99,100]. The importance of the high clinical suspicion is a result of the effectiveness of the treatment.

## Competing interests

The authors declare that they have no competing interests.

## Authors’ contributions

NM, PZ, AS wrote the manuscript, ES, NK, TS, AB and LS provided the pathological images and wrote the legends. AG provided useful insights as an expert pathologist, KZ and CM provided useful insights regarding extrapelvic lesions, PK and NT gathered the necessary references, NC provided and explained the radiologic findings. All authors read and approved the final manuscript.
